# NSK-01105, a Novel Sorafenib Derivative, Inhibits Human Prostate Tumor Growth via Suppression of VEGFR2/EGFR-Mediated Angiogenesis

**DOI:** 10.1371/journal.pone.0115041

**Published:** 2014-12-31

**Authors:** Pengfei Yu, Liang Ye, Hongbo Wang, Guangying Du, Jianzhao Zhang, Yanhua Zuo, Jinghai Zhang, Jingwei Tian

**Affiliations:** 1 School of Life Science and Bio-pharmaceutics, Shenyang Pharmaceutical University, Shenyang, Liaoning 110016, China; 2 State Key Laboratory of Long-acting and Targeting Drug Delivery System, Non-clinical Research Department, Luye Pharma Group Ltd., Yantai, Shandong 264003, China; 3 School of Pharmacy, Yantai University, Yantai, Shandong 264005, China; 4 School of Pharmaceutical Sciences and Institute of Material Medical, Binzhou Medical University, Yantai, Shandong 264005, China; 5 Affiliated Hospital of Medical College of Qingdao University, Qingdao, Shandong 266001, China; Kyung Hee University, Republic of Korea

## Abstract

The purpose of this study is to investigate the anti-angiogenic activities of NSK-01105, a novel sorafenib derivative, in *in vitro*, *ex vivo* and *in vivo* models, and explore the potential mechanisms. NSK-01105 significantly inhibited vascular endothelial growth factor (VEGF)-induced migration and tube formation of human umbilical vein endothelial cells at non-cytotoxic concentrations as shown by wound-healing, transwell migration and endothelial cell tube formation assays, respectively. Cell viability and invasion of LNCaP and PC-3 cells were significantly inhibited by cytotoxicity assay and matrigel invasion assay. Furthermore, NSK-01105 also inhibited *ex vivo* angiogenesis in matrigel plug assay. Western blot analysis showed that NSK-01105 down-regulated VEGF-induced phosphorylation of VEGF receptor 2 (VEGFR2) and the activation of epidermal growth factor receptor (EGFR). Tumor volumes were significantly reduced by NSK-01105 at 60 mg/kg/day in both xenograft models. Immunohistochemical staining demonstrated a close association between inhibition of tumor growth and neovascularization. Collectively, our results suggest a role of NSK-01105 in treatment for human prostate tumors, and one of the potential mechanisms may be attributed to anti-angiogenic activities.

## Introduction

Angiogenesis, new blood vessel formation, plays a pivotal role in the increasing demand for oxygen, nutrients and various growth factors in proliferating tumor cells [1]. Angiogenesis is an essential event in tumor growth, invasion and metastasis, and is tightly regulated by a large number of proangiogenic and anti-angiogenic factors [2]. Among the numerous growth factors and cytokines involved in angiogenesis, vascular endothelial growth factor (VEGF), binding to its receptors, especially VEGFR2, appears to be a key factor in pathological situations that involve tumor neovascularization [3]. Activation of VEGFR2 leads to activation of various downstream signaling proteins, such as focal adhesion kinase (FAK) [4], [5] and endothelial nitric oxide synthases (eNOS) [6], [7], which are involved in several biological processes, including growth, migration and survival of endothelia cells. Several antiangiogenic agents have been approved and been investigated in clinical trials [8]–[12], however, because of the complex process of tumorigenesis and development, a therapeutic agent targeting a single molecular entity might have limited efficacy across a spectrum of tumor types [13], [14].

In recent years, small molecular targeted cancer therapies represent a highly lucrative class of anticancer therapy. Sorafenib is an oral multikinase inhibitor that targets raf kinases as well as a number of receptor tyrosine kinases such as VEGFR2, platelet-derived growth factor receptor, Ret, and c-KIT [8], [15]. In preclinical studies, sorafenib demonstrated promising efficacy against a variety of tumor types based on its inhibitory effect on the Raf/MEK/ERK and angiogenesis pathways. Phase II clinical trials have been performed to determine the efficacy of sorafenib in patients with metastatic or recurrent hormone-refractory adenocarcinoma of the prostate [16]–[18]. Over-expression of EGFR is associated with cancer progression, poor prognosis and development of androgen independence in prostate cancer [19]. Erlotinib, the first-generation EGFR inhibitor, has single-agent activity against various cancer cells, including prostate cancer [20]. Since a series of EGFR inhibitors such as gefitinib, erlotinib, lapatinib and vandetanib were approved for cancer therapy, and thus the 4-aminoquinazoline skeleton has been considered a promising nucleus for antitumor drug development [21]. The combination therapy of anti-EGFR with anti-VEGFR drugs has shown promising results in different tumor models [22], [23]. However, there are few molecular targeted drugs, which focus on dual inhibition of VEGFR and EGFR.

**Figure 1:**
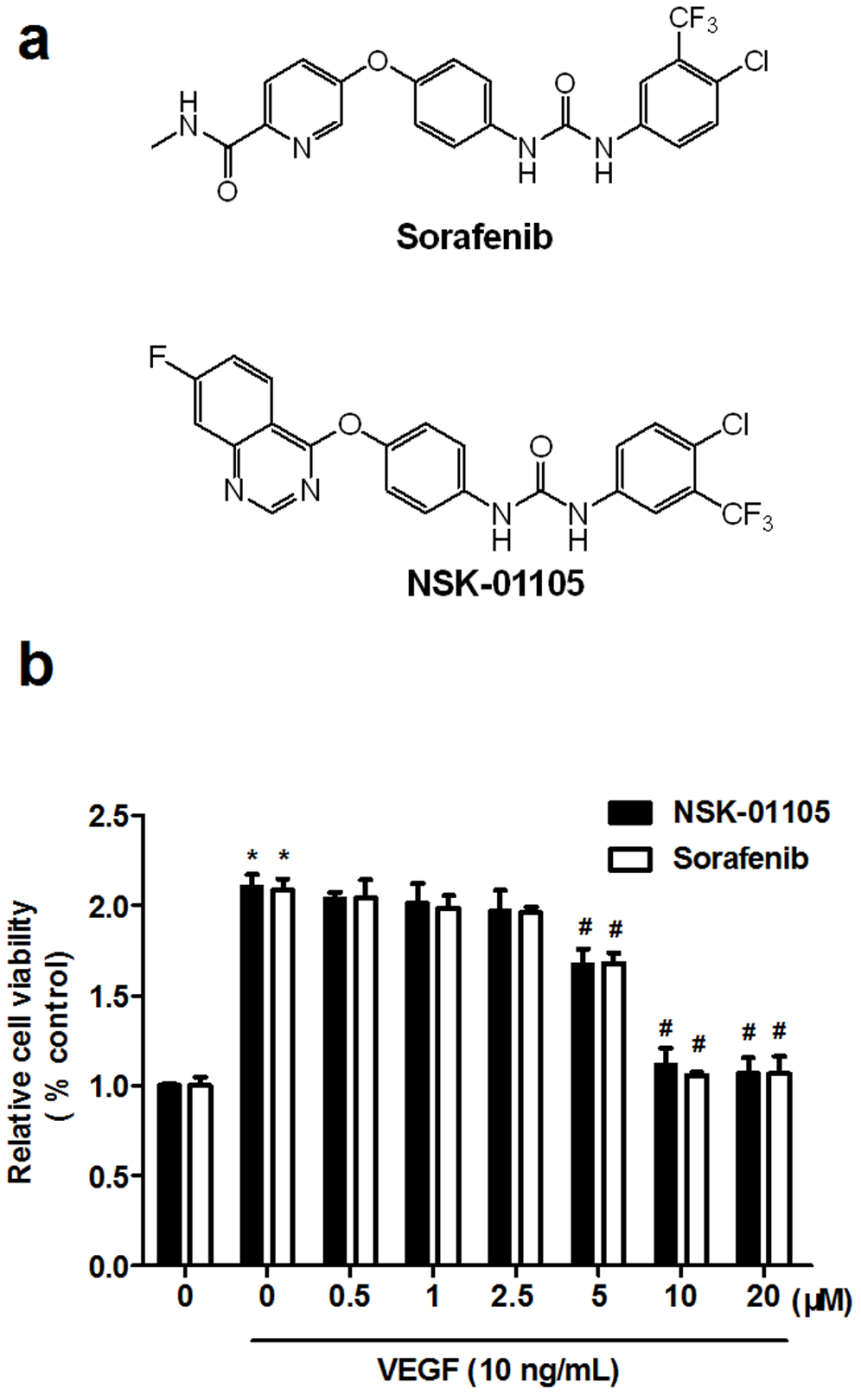
NSK-01105 inhibited VEGF-induced cell viability in HUVECs. (a) Chemical structures of the NSK-01105 and Sorafenib. (b) NSK-01105 inhibited the VEGF-induced viability of endothelial cells. Stimulated with VEGF (10 ng/mL), HUVECs showed a high rate of viability. VEGF-induced viability of HUVECs was significantly inhibited by NSK-01105 and sorafenib at concentrations of 5, 10 and 20 µM for 24 h. Columns, mean; bars, SD (n = 6). *, compared with vehicle controls, *P*<0.05; #, compared with VEGF controls,*P*<0.05.

Based on the structure of sorafenib, we aimed to develop a new series of compounds with enhanced antitumor activities and improved physiological properties. NSK-01105 (Fig. 1a) showed anti-tumor activity in our *in vitro* and *in vivo* screening tests and was selected for further evaluation as a new anti-tumor candidate. The amide group and pyridine ring of sorafenib were replaced by a quinazoline ring, which is considered to be a promising nucleus for EGFR inhibitors. We speculate that NSK-01105 may possess both properties of sorafenib and EGFR inhibitors. In the present study, we investigated the effect of NSK-01105 on the inhibition of tumor specific angiogenesis in *in vitro, ex vivo*, and *in vivo* models in order to support further drug development.

## Materials and Methods

### Ethics Statement

Animals were maintained in laminar-flow cabinets under controlled environment at 25°C on a 12 h light/dark cycle and were provided free access to food and water in strict accordance with the National Institute of Health Guide for the Care and Use of Laboratory Animals (NIH Publications no. 80–23). All animal researches were in compliance with ARRIVE (Animal Research Reporting *In Vivo* Experiments) guidelines (S1 File). All animal protocols were approved by the Ethics Committee of Shenyang Pharmaceutical University (No. 033 in 2012 for Animal Ethics Approval). All surgery was performed under anesthesia, and all efforts were made to minimize suffering.

### Chemicals and Reagents

NSK-01105 (Patent No. CN 2009 1 0026748.8) was synthesized by Nanjing Luye Sike Pharmaceuticals. Sorafenib was purchased from Bayer Pharmaceuticals (West Haven, CT). Compounds were dissolved in DMSO and diluted with RPMI1640 medium to the desired concentrations. VEGF was purchased from Cell Signaling Technology (Beverly, MA). Anti-phospho-VEGFR-2 (Tyr 1059), anti-phospho-EGFR (Tyr 1068) and anti-phospho-FAK (Tyr 397) antibodies were obtained from Cell Signaling Technology (Beverly, MA), and anti-phospho-eNOS (Ser 1177) was purchased from Santa Cruz Biotechnology (Santa Cruz, CA).

### Cell Lines and Cell Culture

Human umbilical vein endothelial cells (HUVECs) were obtained from American Type Culture Collection and cultured in EGM-2 supplemented with 10% fetal bovine serum (FBS). LNCaP and PC-3 cells were purchased from Cell Culture Center of Institute of Basic Medical Sciences, Chinese Academy of Medical Sciences and cultured in RPMI 1640 medium supplemented with 10% FBS. All cells maintained at 37°C in 5% CO_2_.

### Cell Viability Assay

The cell viability rate was evaluated using cytotoxicity assays. Briefly, cells were seeded into 96-well plates and incubated with NSK-01105 or sorafenib at the desired concentrations for 24 h. MTT solution (5 mg/ml) was added to each well and incubated continued for 4 h. DMSO was added to dissolve the MTT formazan product and the absorbance was measured at 570 nm using a Molecular Devices SpectraMax M5 (Molecular Devices, USA). The relative cell viability rates were calculated versus untreated controls. The 50% inhibitory concentration (IC50) values were calculated using the GraphPad Prism 5 (GraphPad Software, Inc., USA).

### Migration Assay

The effects of NSK-01105 on migration of HUVECs were examined by wound-healing and transwell cell migration assays.

Wound-healing assay was performed as previously described [24]. Cells were grown to 90% confluence, and monolayers were scratched with a pipette tip. Cells were washed twice with PBS, and then EGM-2 medium containing VEGF (20 ng/ml), and/or NSK-01105 (0.5, 1 and 2.5 µmol/L) or sorafenib (2.5 µmol/L) were added, and incubated for 16 h. Three randomly selected fields were photographed at the beginning and end of treatment. Migration distance was calculated by Image-Pro Plus software (IPP, Media Cybernetics, USA).

For the transwell cell migration assay, a total of 1.0×10^5^ cells cultured in 200 µL serum-free medium along with the indicated concentrations of VEGF, NSK-01105 or sorafenib were placed in the upper chambers of each insert (8.0 µmol/L pore size, Corning, USA) without matrigel coating. The lower chambers were filled with 0.5 mL of medium containing 10% FBS. After 24 h, the cells were fixed in 4% paraformaldehyde and stained with 0.5% crystal violet. The stained cells were counted under a microscope (Olympus Corp., Japan) in six randomly selected fields and the number of cells was presented relative to untreated controls.

### Endothelial Cell Tube Formation Assay

The formation of HUVEC capillary-like structures on a basement membrane matrix were used to investigate the anti-angiogenic activity of NSK-01105. The 24-well plate was coated with 200 µL matrigel (BD Biosciences) for 30 min at 37°C. HUVECs were seeded on the matrigel bed (1.5 × 10^4^ cells/well) and cultured in EGM-2 medium containing NSK-01105 (0.5, 1 and 2.5 µmol/L) or sorafenib (2.5 µmol/L), in the presence of VEGF (0.3 nmol/L) for 6 h. EGM-2 medium with or without VEGF served as the positive and negative controls, respectively. Tube formation was photographed, and the tube lengths were quantified by IPP software.

### Matrigel Plug Assay

Matrigel (500 µL) containing VEGF at a final concentration of 500 ng/mL, and/or NSK-01105 (0.5, 1 and 2.5 µmol/L) or sorafenib (2.5 µmol/L) was inoculated subcutaneously into the right flank of Balb/c mice. Negative and positive controls were obtained by injecting mice with matrigel in the absence or presence of VEGF. All treatment groups contained four mice. After 10 days, the matrigel plugs were removed and hemoglobin content was determined according to Drabkin's method. The relative hemoglobin content was calculated versus the negative controls.

### Tumor Cells Invasion Assay

The effects of NSK-01105 on the invasion capacity of LNCaP and PC-3 cells were detected by counting the number of cells that migrated through matrigel-coated transwell cell culture chambers. Briefly, an aliquot of 10^5^ cells cultured in 200 µL 2% FBS medium along with NSK-01105 (0.5 and 1 µmol/L) or sorafenib (1 µmol/L) were added into the upper chambers. The lower chambers were filled with 0.5 ml complete medium as chemoattractant. After 24 h incubation, the invaded cells were fixed in 4% paraformaldehyde and stained with 0.5% crystal violet. The stained cells were counted with a microscope and the number of cells was presented relative to untreated controls.

### ELISA Assay for VEGF Protein Levels

For detection of effects of NSK-01105 on the secretion of VEGF in LNCaP and PC-3 cells, the cells were treated for 24 h with NSK-01105 or sorafenib at doses of 0.5, 1, 2.5, 5 or 10 µmol/L. Cell culture supernatants were collected and analyzed using the human VEGF ELISA kit (R&D Systems, Inc., USA) according to the manufacturer's instructions.

### Western Blot Analysis

HUVECs were cultured in growth factor-deprived culture medium for 24 h and then serum starved with serum-free media for 2 h. Cells were exposed to tested articles in serum-free media for 6 h followed by VEGF treatment (30 ng/mL) for 30 min. LNCaP and PC-3 cells were normally cultured and directly treated with NSK-01105 (0.5, 1 or 5 µmol/L) for 6 h. Cells were washed with PBS and lysed with radioimmunoprecipitation assay buffer, and then supernatant was collected followed by centrifugation at 12,000 rpm for 20 min. Cell extracts (40 µg) were subjected to SDS-PAGE, and transferred to nitrocellulose membranes. Membranes were probed with anti-phospho-VEGFR2, anti-phospho-FAK, anti-phospho-eNOS, anti-phospho-EGFR, and horseradish peroxidase-conjugated secondary antibodies, and then detected with an enhanced chemiluminescence system (GE Healthcare Life Sciences) according to the manufacturer's protocol. The β-actin antibody was used as a loading control in western blots for protein normalization. The optical density was quantified by IPP software.

### Human Prostate Tumor Xenograft Models

Male Balb/c *nu/nu* nude mice (5-6 weeks old; purchased from Vital River Laboratory Animal Technology Co., Ltd) were used for *in vivo* experiments. LNCaP cells (2×10^6^ cells per mouse) and PC-3 cells (3×10^6^ cells per mouse) were resuspended in serum-free medium with matrigel basement membrane matrix (BD Biosciences) at a 1∶1 ratio, and then subcutaneously injected into the right flank of each animal (6 animals/group). After tumors grew to about 120 mm^3^ in size, mice were treated orally once daily with NSK-01105 or sorafenib at a dose of 60 mg/kg for 14 days. The dose was chosen on based on studies of sorafenib in various cancer models [8]. Tumor dimensions and body weights were recorded twice weekly. The tumor weight was calculated using the formula (*l*×(*w*)^2^)/2, where *l* was the longest dimension of the tumor and *w* was the width. The inhibition rate (IR) of tumor growth was calculated by the following formula: IR (%)  =  [(*A*-*B*)/*A*]×100, where *A* and *B* were the mean tumor weight in the vehicle control and treatment groups, respectively.

### Immunohistochemical Staining and Quantification of Microvessels

All animals were sacrificed under anesthesia (ketamine 100 mg/kg plus xylazine 20 mg/kg, im) after the last treatment and tumors were harvested and fixed in paraformaldehyde for 24 h for immunohistochemical staining assay. Paraffin sections were incubated with anti-CD31 (Cell Signaling Technology) antibody overnight at 4°C, and then incubated with a biotinylated secondary antibody and avidin-biotin-peroxidase complex for 30 min at 37°C. Immunoreactive signals were developed with 0.05% diaminobenzidine in Tris-HCl buffer (0.1 mol/L, pH 7.6) containing 0.03% H_2_O_2_.

The tissue sections were viewed at ×100 magnification and images were captured with a digital camera. Four fields per section were randomly analyzed and areas of CD31 positive objects were quantified using the IPP software. Percentage of microvessel area (MVA, %) in each field was calculated as follows: (area of CD31-positive objects/measured tissue area) ×100%. Mean values of MVA in each group were calculated from four tumor samples.

### Statistical Analysis

Statistical analysis was performed with the PASW 18.0 software package. Data points were presented as mean ± S.D., and analyzed using one-way analysis of variance followed by Dunnett's test. For all tests, *P<*0.05 was considered statistically significant.

## Results

### NSK-01105 Inhibited HUVEC Viability

Endothelial cell viability is an essential step in angiogenesis. The effect of NSK-01105 on HUVECs viability was examined by cytotoxicity assay. As shown in Fig. 1b, stimulated with VEGF (10 ng/mL), HUVECs showed a high viability rate. Treatment with NSK-01105 or sorafenib at 5, 10 and 20 µmol/L for 24 h significantly inhibited VEGF-induced viability of HUVECs in a dose-dependent manner. There were no significant difference on HUVEC viability between NSK-01105 and sorafenib. Both compounds were non-toxic to HUVECs at concentrations of 0.5, 1 and 2.5 µmol/L, and these concentrations were used for further *in vitro* and *ex vivo* experiments.

### NSK-01105 Inhibited HUVEC Migration

**Figure 2:**
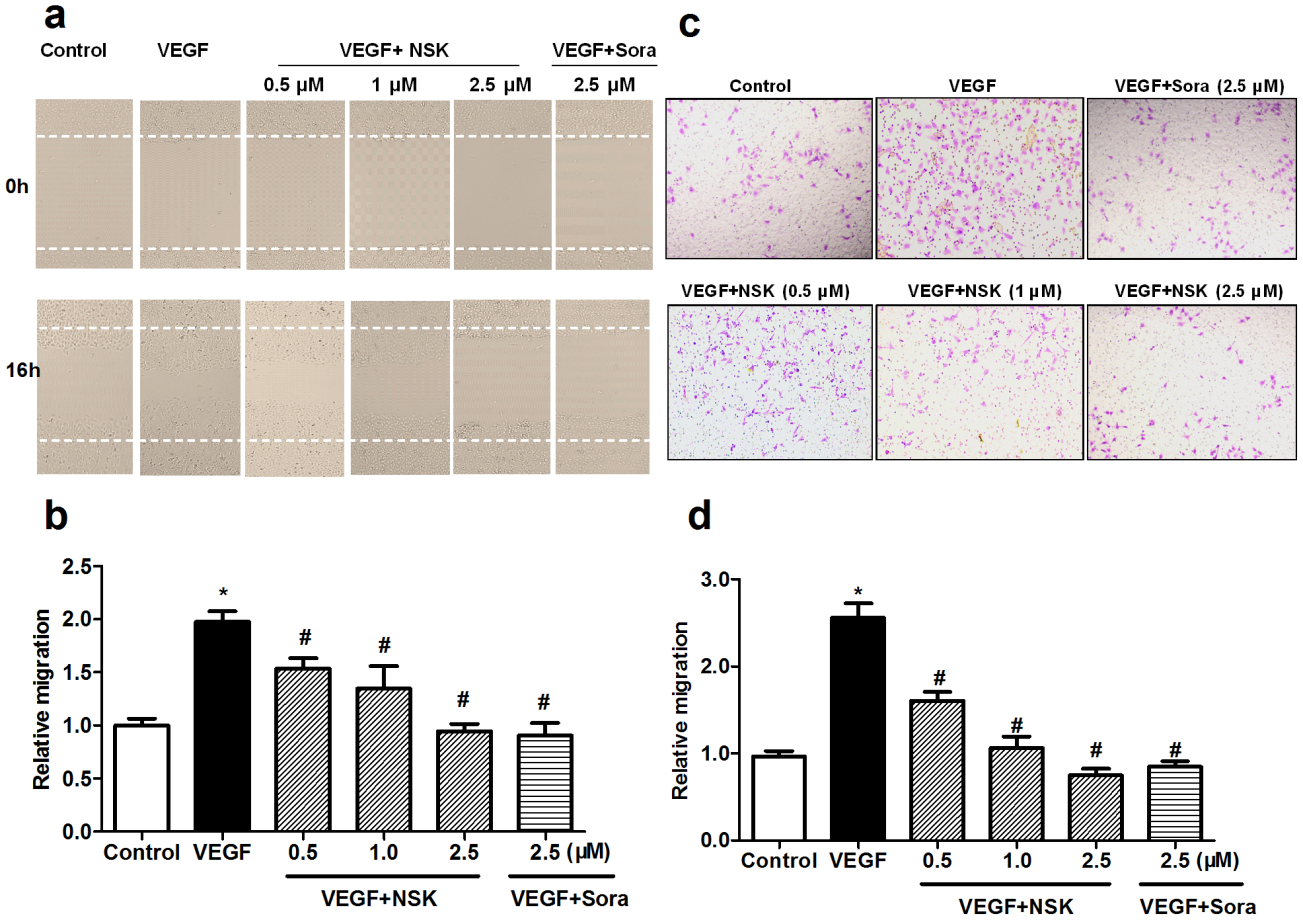
NSK-01105 inhibited VEGF-induced migration of HUVECs. (a, b) NSK-01105 inhibited HUVECs migration by wound-healing assay. Cells were starved to inactivate cell proliferation and then wounded by pipette tips. Stimulated with VEGF, large numbers of HUVECs migrated into the clear area, whereas, as potent as sorafenib, NSK-01105 significantly inhibited the VEGF-induced migration at 0.5, 1 and 2.5 µM. Migration distances were calculated by IPP software. (c, d) NSK-01105 inhibited HUVECs migration by transwell cell migration assay. Cells were cultured in serum-free medium along with the indicated concentrations of VEGF, NSK-01105 and/or sorafenib in the upper chambers and the lower chambers were filled with 10% FBS medium. Stimulated with VEGF, large numbers of HUVECs migrated into the lower surface of the polycarbonate, whereas NSK-01105 and sorafenib significantly inhibited the VEGF-induced migration. Cells were counted under a microscope. Columns, mean; bars, SD (n = 4). *, compared with vehicle controls, *P*<0.05; #, compared with VEGF controls, *P*<0.05.

Migration is also essential in angiogenesis. The effect of NSK-01105 on the chemotactic motility of HUVECs was determined by wound-healing assay and transwell cell migration assay. Stimulated with VEGF, large numbers of HUVECs migrated into the clear area (Fig. 2a and 2b) and the lower surface of the polycarbonate (Fig. 2c and 2d). Notably, as potent as sorafenib, the VEGF-induced migration was significantly inhibited by NSK-01105 at 0.5, 1 and 2.5 µmol/L. These concentrations proved to be non-toxic by cytotoxicity assay and thus the inhibitory effect could not be attributed to cytotoxicity.

### NSK-01105 Inhibited Tube Formation and *ex vivo* Angiogenesis in Matrigel Plug Assay

**Figure 3:**
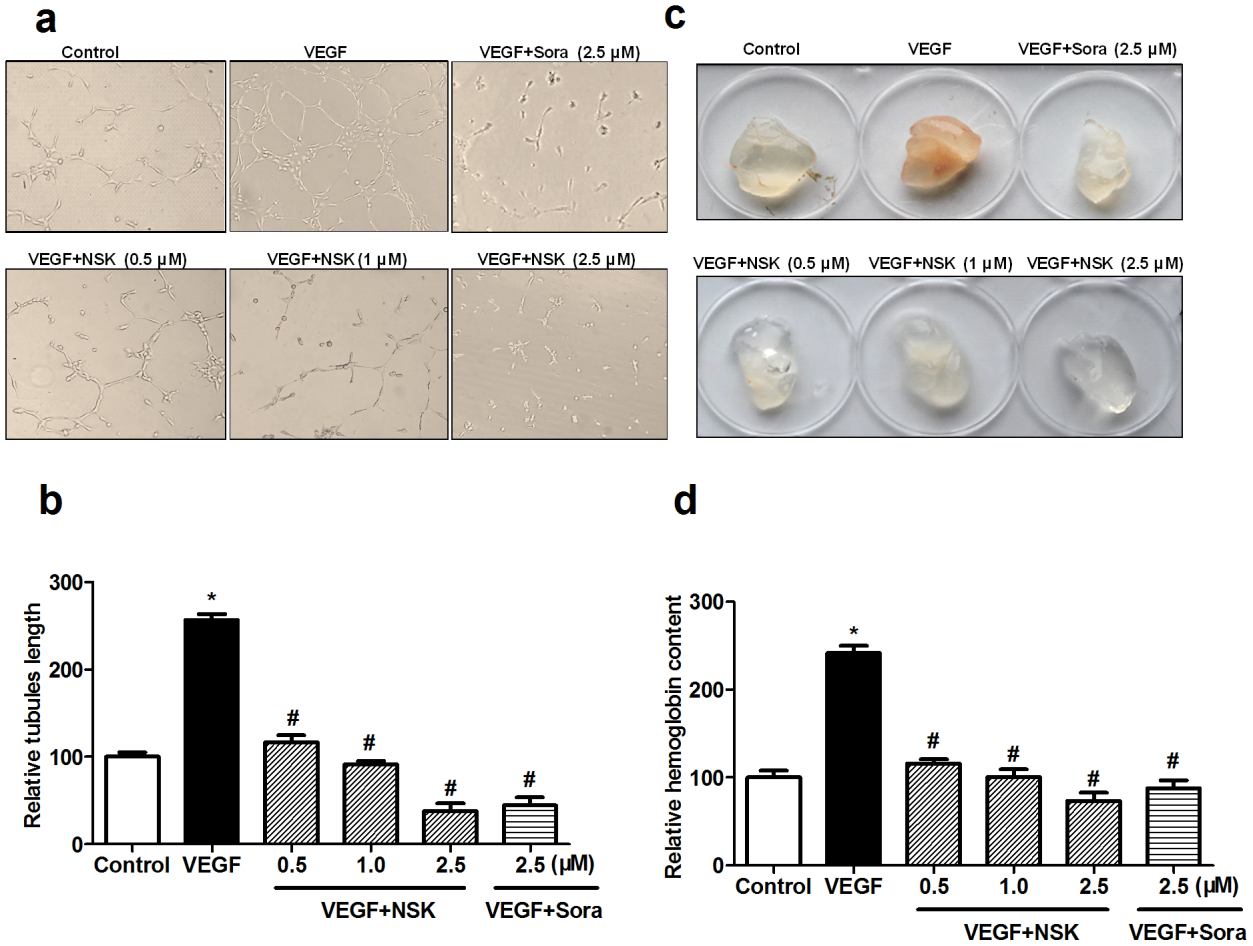
NSK-01105 inhibited the tube formation of HUVECs and *ex vivo* angiogenesis in matrigel plug assay. (a, b) NSK-01105 inhibited the tube formation of HUVECs. HUVECs were seeded on the matrigel bed and treated with various dilutions of NSK-01105 with or without VEGF (0.3 nmol/L) for 24 h. Tubular structures were photographed and the tube lengths were quantified by IPP software. (c, d) NSK-01105 inhibited *ex vivo* angiogenesis by matrigel plug assay. The matrigels, which contained VEGF (500 ng/mL), and NSK-01105 or sorafenib, were inoculated subcutaneously into the right flank of mice. After 10 days, matrigel plugs were removed and hemoglobin content was determined according to the Drabkin's method. Columns, mean; bars, SD (n = 4). *, compared with vehicle controls, *P*<0.05; #, compared with VEGF controls, *P*<0.05.

Tube formation of endothelial cells is one of the critical steps to the formation of new blood vessels [25]. Little tube formation was observed when cells were cultured only in medium. However, tube formation significantly increased in the presence of VEGF and significantly decreased upon treatment with NSK-01105 at 0.5, 1 and 2.5 µmol/L and sorafenib at 2.5 µmol/L (Fig. 3a and 3b).

To further confirm the anti-angiogenesis effects of NSK-01105, matrigel plug assay was performed. As shown in Fig. 3c, VEGF-induced angiogenesis in the matrigel plug was significantly inhibited by NSK-01105 and sorafenib. Relative hemoglobin content was also significantly decreased in NSK-01105 or sorafenib treated matrigel plug mice by Drabkin's method (Fig. 3d). These data indicated that, as potent as sorafenib, NSK-01105 effectively inhibited tube formation of endothelial cells and angiogenesis in the matrigel plug.

### NSK-01105 Inhibited the Activation of VEGFR2-Mediated Signal Pathways

**Figure 4:**
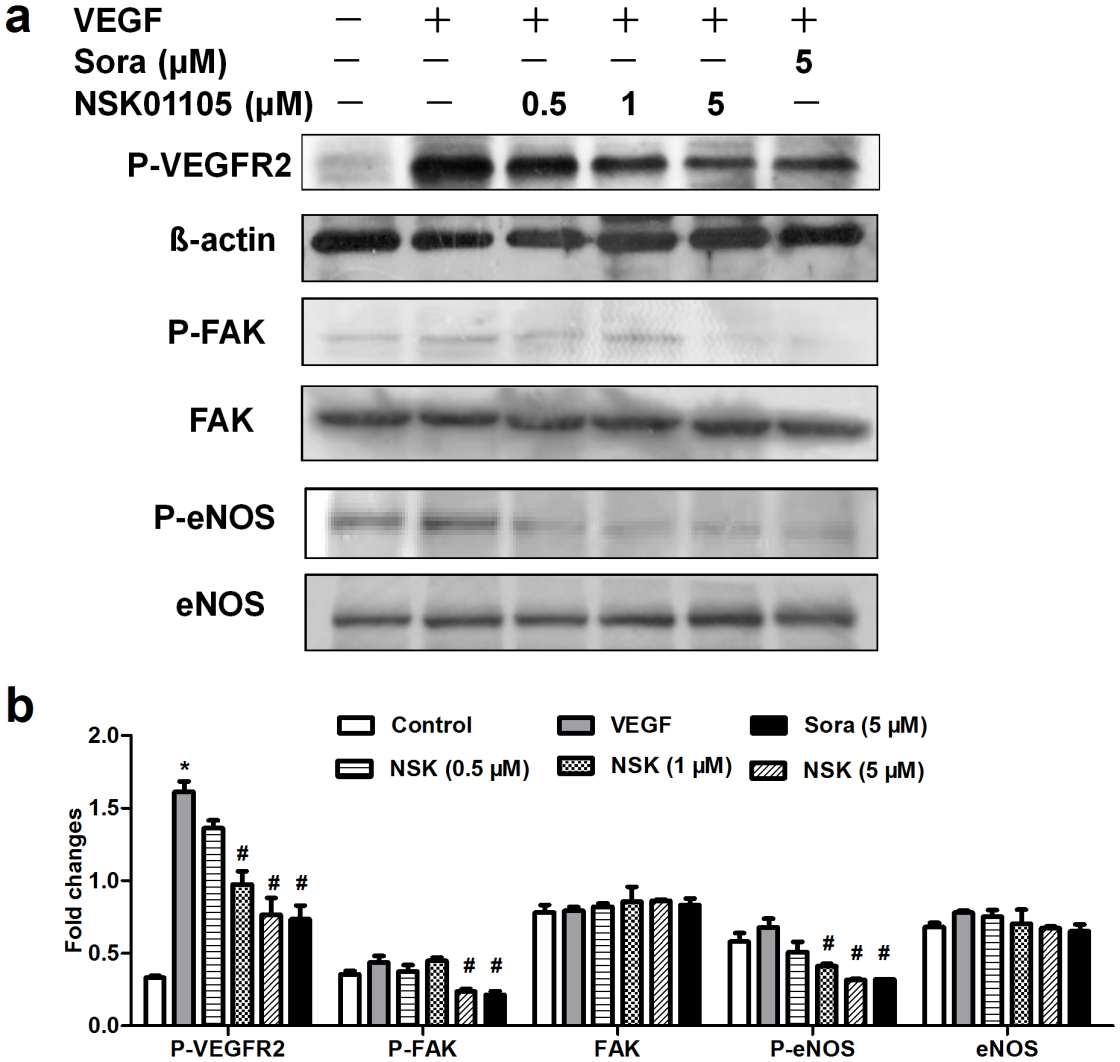
NSK-01105 inhibited the activation of VEGFR2-mediated signaling pathways in HUVECs. VEGF stimulated VEGFR-2 autophosphorylation in HUVECs. (a) NSK-01105 and sorafenib suppressed the activation of VEGFR2 and its downstream key kinases FAK and eNOS at 5 µM in HUVECs. (b) The ratios of the optical density between target molecules and β-actin. The optical density was quantified by IPP software. Columns, mean; bars, SD (n = 3). *, compared with vehicle controls, *P*<0.05; #, compared with VEGF controls, *P*<0.05.

To explore the potential molecular mechanism of antiangiogenic properties induced by NSK-01105, several downstream key kinases, such as FAK and eNOS, which are involved in VEGFR2-mediated signaling pathways, were examined. As shown in Fig. 4a and 4b, VEGF (30 ng/mL) dramatically induced VEGFR2 autophosphorylation, which was blocked by NSK-01105 and sorafenib at a concentration of 5 µmol/L. The phosphorylation levels of FAK and eNOS were also inhibited by NSK-01105 and sorafenib, while total FAK and eNOS levels were unchanged. These data suggested that the anti-angiogenic activity induced by NSK-01105 in endothelial cells was through down-regulation of the activation of VEGFR2-mediated signal pathways, which was similar to the effect of sorafenib.

### NSK-01105 Inhibited Proliferation and Invasion of Prostate Cancer Cells

**Figure 5:**
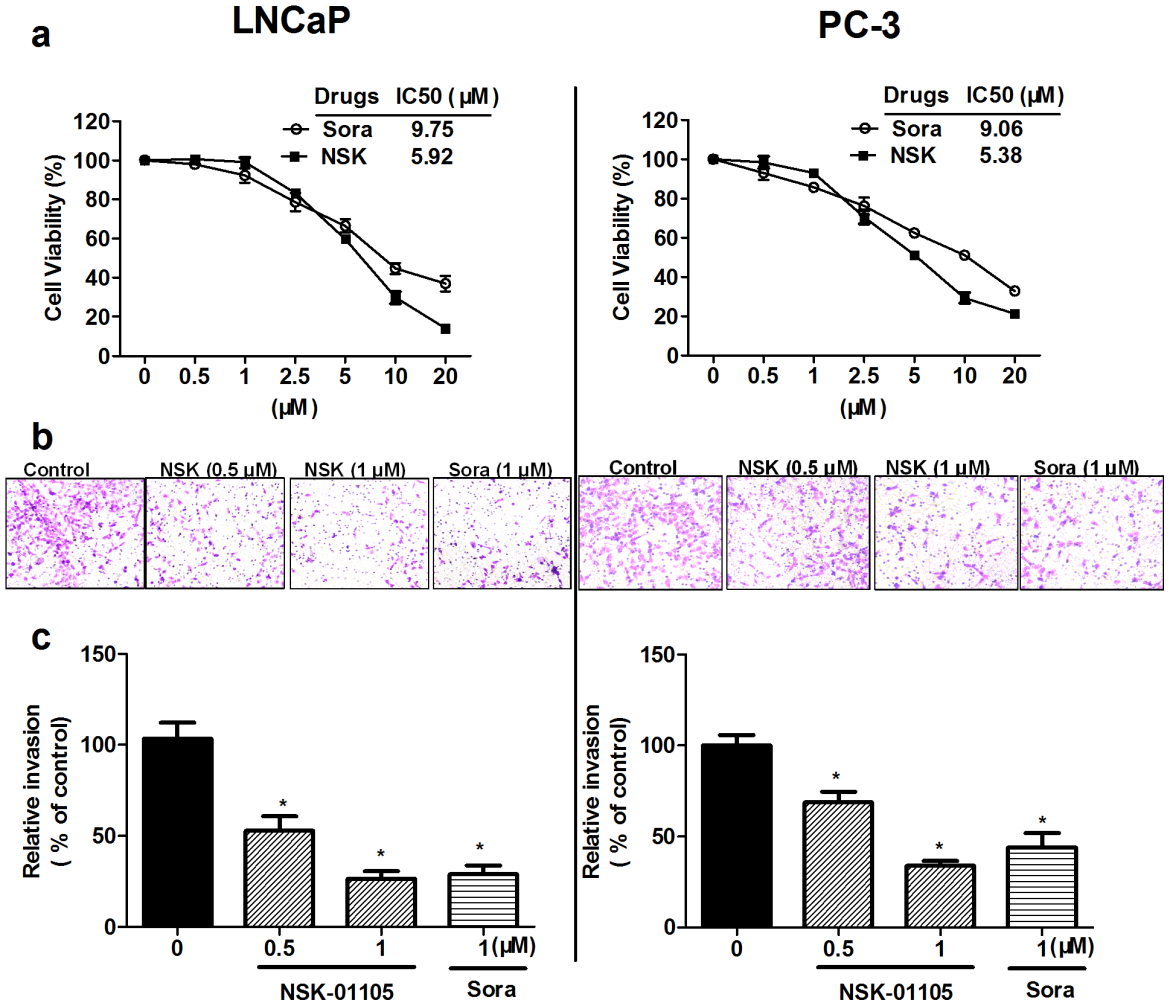
NSK-01105 inhibited cell viability and invasion in LNCaP and PC-3 cells. (a) NSK-01105 inhibited cell viability in prostate cancer cells. Both LNCaP and PC-3 cell viability was significantly inhibited by NSK-01105 and sorafenib in a dose dependent manner. NSK-01105 was more active against both prostate cancer cells compared with sorafenib for 24 h, with the IC50 values of 5.92 and 5.38 µmol/L, respectively. Points, mean; bars, SD (n = 6). (b, c) NSK-01105 inhibited cell invasion in LNCaP and PC-3 cells. Invasion assay in matrigel-coated transwell chamber was performed to confirm the invasion effects of NSK-01105 in both prostate cancer cell lines. NSK-01105 suppressed the invasion of both cell lines at non-toxic concentrations of 0.5 and 1 µM. Columns, mean; bars, SD (n = 4). *, compared with vehicle controls, *P*<0.05.

The effect of NSK-01105 on prostate cancer cell proliferation is shown in Fig. 5a. Both androgen-sensitive LNCaP cells and androgen-independent PC-3 cells were significantly inhibited by NSK-01105 at concentrations of 2.5–20 µmol/L in a dose-dependent manner. Additionally, NSK-01105 was more active against both prostate cancer cells compared with sorafenib for 24 h, with the IC50 values of 5.92 and 5.38 µmol/L, respectively.

Cancer cell invasion is one of the important and characteristic steps in cancer metastasis. Therefore, invasion assays in matrigel-coated transwell chamber were performed to confirm the invasion effects of NSK-01105 in both prostate cancer cell lines. The invasion of both cell lines were significantly suppressed by NSK-01105 at low concentrations of 0.5 µmol/L and 1 µmol/L, with a relative invasion rate of 60.9% and 49.8% in LNCaP cells, and 77.0% and 43.7% in PC-3 cells, respectively (Fig. 5b and 5c). NSK-01105 was non-toxic to LNCaP and PC-3 cells at concentrations of less than 1 µmol/L, and thus the inhibitory effect could not be attributed to cytotoxicity.

### NSK-01105 Inhibited VEGF Protein Secretion and VEGFR-2 and EGFR Activation

**Figure 6:**
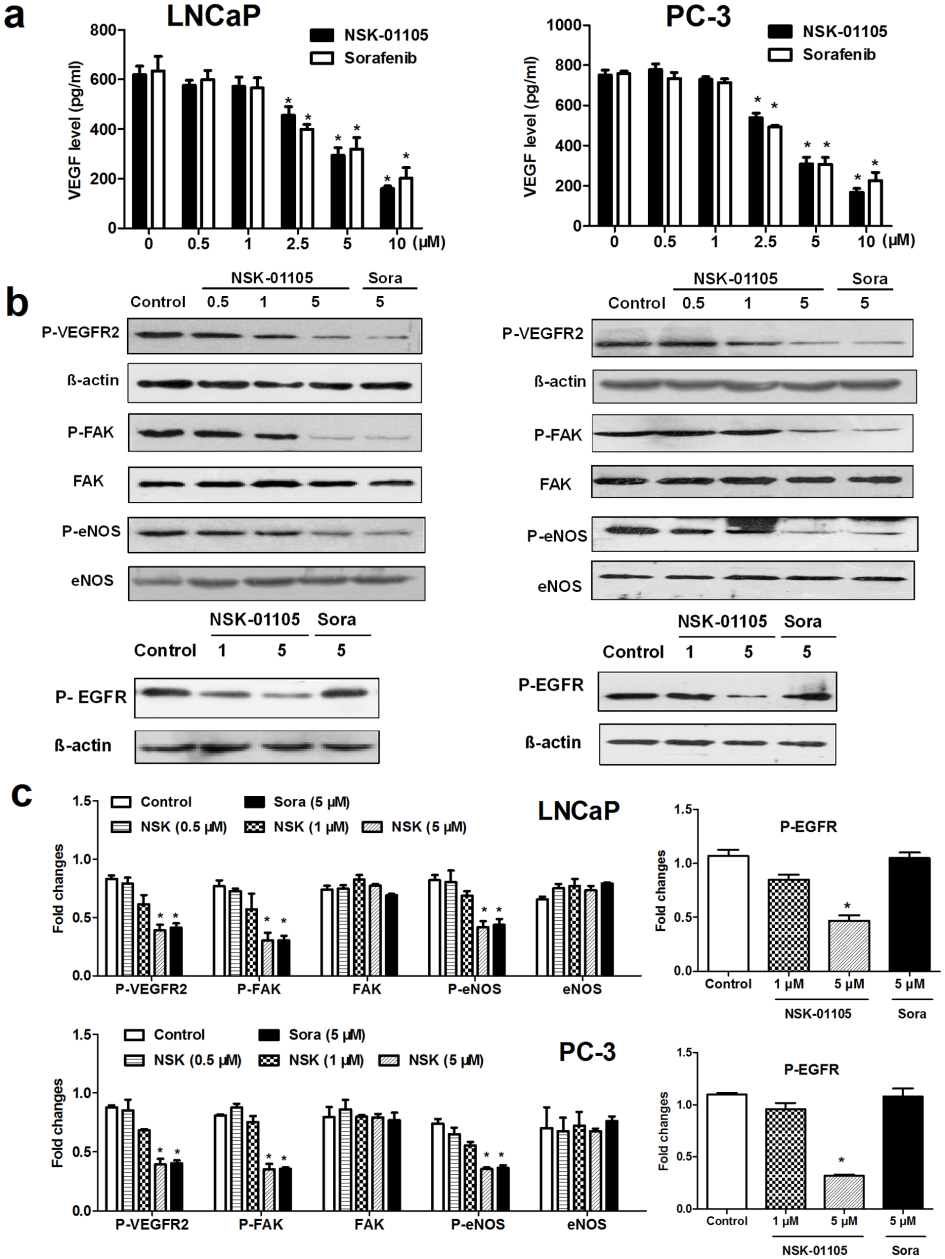
NSK-01105 inhibited VEGF secretion and the activation of VEGFR2-mediated signaling pathways in prostate cancer cells. (a) NSK-01105 inhibited VEGF secretion in prostate cancer cells. VEGF levels were estimated by ELISA method. NSK-01105 and sorafenib suppressed VEGF secretion in both cell lines at concentrations of 2.5, 5 and 10 µM. Columns, mean; bars, SD (n = 3). *, compared with vehicle controls, *P*<0.05. (b) NSK-01105 and sorafenib suppressed the activation of VEGFR2 and its downstream key kinases FAK and eNOS at 5 µM in both prostate cancer cells. NSK-01105 suppressed the phosphorylation of EGFR, while sorafenib had little effect on EGFR activation at 5 µM in both prostate cancer cell lines. (c) The ratios of the optical density between target molecules and β-actin. The optical density was quantified by IPP software. Columns, mean; bars, SD (n = 3). *, compared with vehicle controls, P <0.05.

VEGF is the main stimulatory factor in angiogenesis and is highly secreted by LNCaP and PC-3 cells [26]. ELISA analysis showed that, as potent as sorafenib, NSK-01105 reduced the secretion of VEGF at concentrations of 2.5–10 µmol/L in both LNCaP and PC-3 cells in a dose-dependent manner (Fig. 6a).

To further determine whether NSK-01105 can block activation of the VEGFR-2/FAK/eNOS pathway in prostate cancer cells, western blot analysis was performed. As shown in Fig. 6b and 6c, the phosphorylated levels of VEGFR2, FAK and eNOS were all blocked by NSK-01105 at 5 µmol/L.

The quinazoline skeleton is considered to be a promising nucleus for EGFR inhibitors [27]. We further investigated whether NSK-01105 also affected the activities of EGFR. NSK-01105 suppressed the phosphorylation of EGFR, while sorafenib had little effect on the activation of EGFR.

These results suggested that NSK-01105 exhibited the potential antiangiogenic activity on prostate cancer, by reducing VEGF secretion and inhibiting the activation of both VEGFR-2 and EGFR.

### NSK-01105 Inhibited Tumor Growth and Tumor Angiogenesis

**Figure 7:**
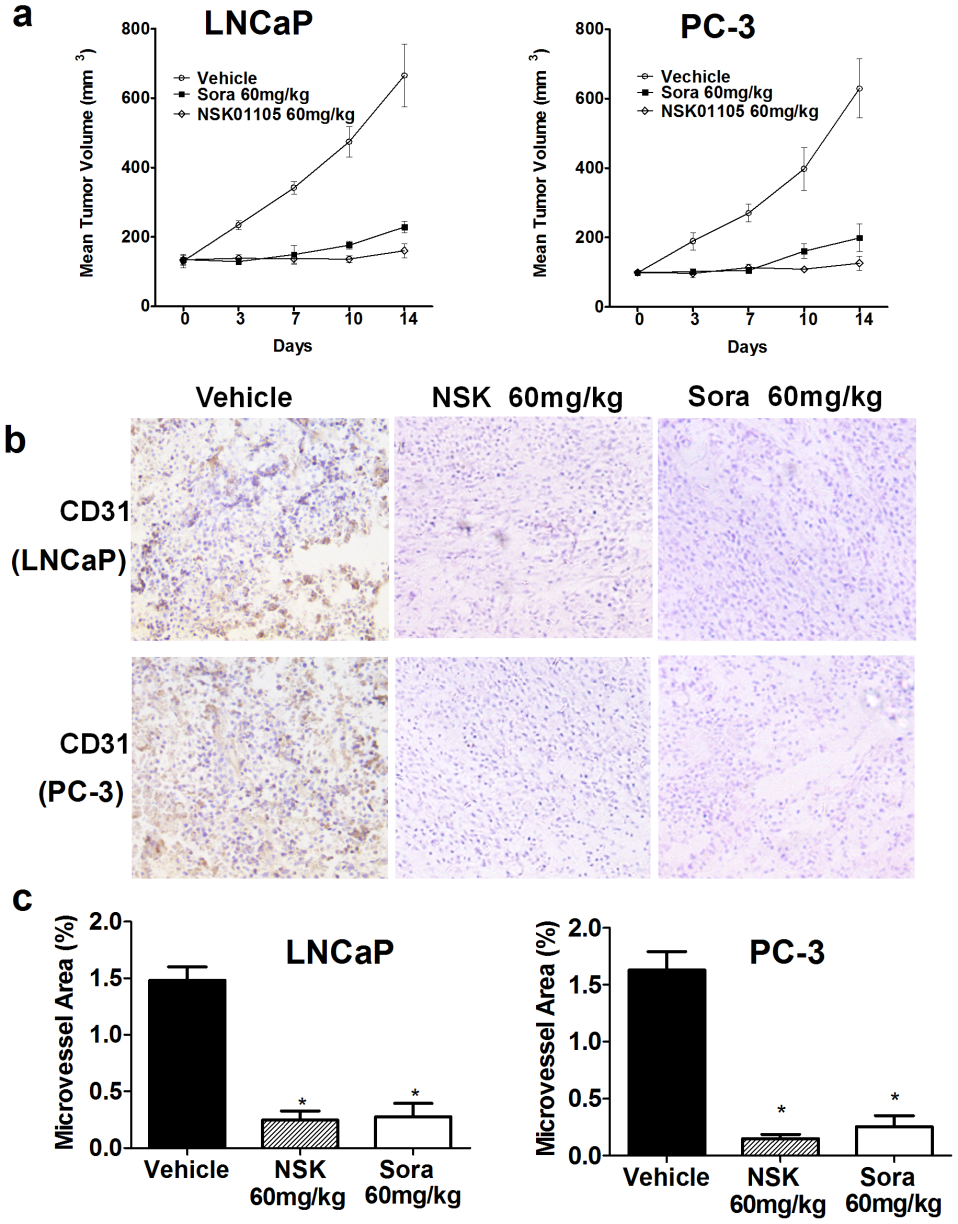
NSK-01105 produced robust anti-cancer effects and inhibited neovascularization in prostate tumor xenograft models. (a) NSK-01105 inhibited tumor growth in xenograft models. LNCaP cells (2×10^6^) or PC-3 cells (3×10^6^) were implanted into the right flank of each animal. NSK-01105 was administered orally once daily for 14 days at a dose of 60 mg/kg. NSK-01105 had satisfactory inhibition effect against LNCaP and PC-3 tumor growth compared with sorafenib at a dose of 60 mg/kg. Points, mean tumor volume; bars, SD (n = 6). (b) CD31-positive objects were observed in the tumor tissue (brown-colored objects). (c) The number and area of microvessels were measured, and percent area microvessel was calculated in each group. Columns, mean; bars, SD (n = 4) *, *P*<0.05.

Two xenograft prostate tumor models were used to investigate the effects of NSK-01105 on tumor growth and angiogenesis. Mice bearing 120 mm^3^ tumors were treated orally at 60 mg/kg, once daily for 14 days. As shown in Fig. 7a and Table 1, NSK-01105 and sorafenib produced durable partial tumor regression with IRs of 76.35% and 67.57% in LNCaP models and 71.43% and 60.21% in PC-3 models, respectively, but complete tumor regressions were not achieved during treatment because of the persistence of matrigel even if the cells were completely eliminated. Additionally, some side effects were observed in the sorafenib group, including unkempt appearance, weight loss or even death. By comparison, animals in the NSK-01105 group only showed slight weight loss. All these data suggested that NSK-01105 had satisfactory inhibition effect against LNCaP and PC-3 tumor growth with fewer side effects comparing with sorafenib.

**Table 1:** The effect of NSK-01105 and sorafenib in prostate cancer xenograft models.

Tumor	Group	Dosage (mg/kg/day)	No. of animals (n)	Body weight (g)		Tumor weight (g)	IR (%)
			Begin/End	Begin	End		
LNCaP	Control		6/6	17.11±2.68	26.44±2.75	1.48±0.20	
	Sorafenib	60	6/4	18.04±2.20	24.08±3.44	0.48±0.12*	67.57
	NSK-01105	60	6/6	17.86±2.89	26.04±2.84	0.35±0.09*	76.35
PC-3	Control		6/6	18.08±2.61	27.28±2.65	0.98±0.12	
	Sorafenib	60	6/6	17.88±2.22	25.42±2.52	0.39±0.15*	60.21
	NSK-01105	60	6/6	17.92±2.20	26.92±3.02	0.28±0.11*	71.43

*, *p*<0.05, compared with control. Significant difference was calculated by one-way analysis of variance.

Angiogenesis provides necessary nutrients and oxygen for tumor development, growth and metastasis. To further explore whether NSK-01105 affects tumor growth by suppressing angiogenesis, we evaluated tumor MVA in tumor samples. As shown in Fig. 7b, the area of CD31 positive cells observed with NSK-01105 treatment was significantly reduced compared with the vehicle-treated group. NSK-01105 and sorafenib produced 80–90% inhibition of MVA relative to vehicle-treated animals at a dose of 60 mg/kg (Fig. 7c).

Taken together, our data showed that NSK-01105 demonstrated robust antitumor efficacy in human prostate tumor xenograft models. Inhibition of tumor angiogenesis should be one of the potential mechanisms.

## Discussion

Prostate cancer is the most common cancer and the second leading cause of cancer-related mortality with an estimated 240,000 new diagnoses in the United States in 2013 [28]. The incidence of prostate cancer increases exponentially with advancing age. Prostate cancer is also potentially associated with continued increases in the prevalence of risk factors such as physical inactivity, diet with high fat intake and less vegetables [29], [30]. Consequently, there is a high incentive for drug development because of the large number of people affected by this disease. Standard therapy approaches include surgical castration, radiation therapy, hormonal therapy (including administration of luteinizing hormone-releasing hormone analogues and antiandrogens) and cytotoxics [28], [31]. Hormonal therapy drugs dominate the treatment of hormone sensitive prostate cancer, and adequate disease control can be achieved for a number of years. Most prostate cancers are initially hormone-dependent, and the effects of anti-hormonal drugs are primarily centered on hormone deprivation for patients with advanced disease. However, nearly all patients eventually progress to develop castration-resistant prostate cancer (CRPC) and most anti-hormonal therapies are ineffective at this stage [23]. Additionally, companies may be reluctant to develop anti-hormonal drugs because of the intense competition in this saturated section of the market. Docetaxel is the first-line standard-of-care chemotherapy choice for metastatic CRPC. However, apart from a relatively short extension of survival, approximately 50% prostate cancer patients initially do not respond to treatment and are exposed to significant toxicity [32]. Furthermore, companies may have recognized the difficulty of developing a cytotoxic drug capable of capturing docetaxel's market share. This is especially true given that patent expiry has enabled the launch of cheaper generic docetaxel, which is an attractive therapy for physicians looking to economize.

Currently, the interest of drug discovery has shifted to small molecular targeted therapies including high tumor specificity and low general toxicity [33]. Angiogenesis is a hallmark of a variety of tumor types, thus the development of angiogenesis inhibitors seems promising for effective therapeutics. Angiogenesis is also important in prostate cancer, and VEGF family members have emerged as viable targets for prostate cancer therapy [34], [35]. VEGF and its receptor VEGFR2 play a pivotal role in the regulation of tumor vessel formation [36]. Therefore, VEGFR2, as the main receptor of VEGF's pro-angiogenic signal transducer, is a promising molecular target for anti-angiogenic therapy. Evidence has been shown that inhibitors of VEGF and VEGFR2 reduce endothelial cell proliferation, migration and survival that lead to regression of vessel density and decrease vascular permeability, thereby slowing tumor growth [37], [38]. Currently, a number of VEGFR2 inhibitors are under clinical trials (ramucirumab, cediranib) and several have been approved (sunitinib, sorafenib). As predicted, based on the similarity of the structure of NSK-01105 with its mother compound sorafenib, NSK-01105 retained the inhibitory effects on VEGFR2.

EGFR regulates cell proliferation, differentiation, angiogenesis and survival. It is expressed at high levels in prostate cancer and over-expression of EGFR is associated with cancer progression, poor prognosis and development of androgen independence [19]. Erlotinib, a selective tyrosine kinase inhibitor for EGFR, have reported benefits prostate cancer patients in clinical studies [20]. However, administration of gefitinib alone, another EGFR inhibitor, is ineffective and combined administrations of gefitinib and radiation therapy or docetaxel have limited therapeutic effects [39]–[41]. The combination therapy of anti-EGFR with anti-VEGFR drugs has shown promising results in different tumor models [22], [23]. Interestingly, NSK-01105 exhibited dual inhibition of VEGFR2 and EGFR in both postate cancer cells.

Sorafenib has been successfully approved for treatment of renal cell carcinoma (2005), hepatocellular carcinoma (2007) and differentiated thyroid carcinoma. However, it has been reported that sorafenib may cause various hematological and cutaneous side effects [42], and even skin cancer [43] in clinical treatment. Thus we chose sorafenib as the lead compound in order to obtain novel tyrosine kinase inhibitors with higher activity and lower side effect. Based on the analysis of structure activity relationship of sorafenib and its analogs, we decided to keep the urea group and transform the amide group and pyridine ring into quinazoline ring. Meanwhile, we added fluoro substituent to enhance electronegative environment. We speculate that the new compound could retain some properties of sorafenib and introduce some properties of EGFR inhibitors. NSK-01105 appears to be similar to, but may have advantages over sorafenib due to the dual inhibition of VEGFR2 and EGFR activation in laboratory and animal tests. However, like sorafenib, the absorption of NSK-01105 is difficult because of its poor water solubility. Further developments have been performed to improve solubility and dissolution of NSK-01105 in the salt formation in order to improve drug absorption and subsequently enhance antitumor activities *in vivo*.

In this study, we confirmed that, as potent as sorafenib, NSK-01105 significantly inhibited VEGF induced migration and tube formation of HUVECs at non-cytotoxic concentrations and inhibited angiogenesis in matrigel plug assay. NSK-01105 also inhibited viability and invasion of LNCaP and PC-3 cells. We further demonstrated that the antiangiogenic activities of NSK-01105 were associated with reducing the VEGF-induced phosphorylation of VEGFR-2 and the activation of EGFR. Furthermore, NSK-01105 exhibited satisfactory inhibition effect on prostate cancer cells proliferation *in vitro* and *in vivo*, and with fewer side effects compared with sorafenib in both xenograft models.

In conclusion, NSK-01105 demonstrated robust antitumor efficacy in human prostate tumor cells. One of the potential mechanisms may be attributed to VEGFR2 and EGFR-mediated angiogenesis. As a novel sorafenib derivative, NSK-01105 appears to be a promising orally active anticancer drug and deserves further investigation.

## Supporting Information

S1 File **The reporting of animal experiments follow the ARRIVE guidelines.** The ARRIVE checklist.
(DOCX)
