# Supplementary material for: NSK-01105, a Novel Sorafenib Derivative, Inhibits Human Prostate Tumor Growth via Suppression of VEGFR2/EGFR-Mediated Angiogenesis
Source: PLoS One. 2014 Dec 31;9(12):e115041. doi: 10.1371/journal.pone.0115041 (PMC4281216; doi:10.1371/journal.pone.0115041)
Supplement: S1 File — The reporting of animal experiments follow the ARRIVE guidelines. The ARRIVE checklist. (DOCX) [file pone.0115041.s001.docx]

| **File S1. The ARRIVE (Animal Research: Reporting of In Vivo Experiments) Checklist** | |
| --- | --- |
| ITEM RECOMMENDATION | |
| Title | NSK-01105, a Novel Sorafenib Derivative, Inhibits Human Prostate Tumor Growth via Suppression of VEGFR2/EGFR-Mediated Angiogenesis. |
| Abstract | BACKGROUND AND PURPOSE: Angiogenesis is regarded as an essential event in tumor growth, invasion and metastasis, and it is tightly regulated by a large number of proangiogenic and antiangiogenic factors. Our aim was to evaluate the effects of NSK-01105, a novel sorafenib derivative, on the inhibition of tumor specific angiogenesis in human prostate tumor xenograft models in order to support further drug development.  EXPERIMENTAL APPROACH: Male Balb/c *nu/nu* nude mice (6 animals/group) were used for *in vivo* experiments. LNCaP and PC-3 cells were subcutaneous injected into the right flank of each animal. After tumors grew to about 120 mm^3^ in size, mice were treated orally once daily with vehicle (saline and dimethyl sulfoxide, DMSO), NSK-01105 (60 mg/kg) or sorafenib (60 mg/kg) for 14 days. Tumor dimensions and body weights were recorded twice weekly. Animals were sacrificed under anesthesia after the last treatment and tumors were harvested and fixed in paraformaldehyde for immunohistochemical staining assay. The areas of CD31 positive objects were quantified. NSK-01105 demonstrated robust antitumor efficacy in both xenograft models and inhibition of tumor angiogenesis should be one of the potential mechanisms. |
| INTRODUCTION | |
| Background | Prostate cancer is the most common cancer and the second leading cause of cancer-related mortality with an estimated 240,000 new diagnoses in the United States in 2013. Angiogenesis is a hallmark of variety of tumor types including human prostate cancer, and the development of angiogenesis inhibitors seemed logical and relevant. Sorafenib is an oral multikinase inhibitor that targets raf kinases as well as a number of receptor tyrosine kinases such as VEGFR2, platelet-derived growth factor receptor, Ret, and c-KIT. Based on the structure of sorafenib, we are interested to synthesize NSK-01105. The amide group and pyridine ring of sorafenib are replaced by quinazoline ring, which is considered to be a promising nucleus for EGFR inhibitors. We investigate the antitumor and antiangiogenic activities of NSK-01105 and explore the potential mechanisms in human tumor xenograft models. |
| Objectives | The objectives of this study were to determine whether NSK-01105, a novel sorafenib derivative, inhibits tumor growth and tumor specific angiogenesis. |
| METHODS | |
| Ethical statement | Animals were maintained in laminar-flow cabinets under controlled environment at 25 °C on a 12-hours light/dark cycle and provided free access to food and water in strict accordance with the National Institute of Health Guide for the Care and Use of Laboratory Animals (NIH Publications no. 80-23)*.* All animal protocols were approved by the Ethics Committee of Shenyang Pharmaceutical University (No. 033 in 2012 for Animal Ethics Approval). Animals were sacrificed under anesthesia and all efforts were made to minimize suffering. |
| Study design | Three groups of 6 mice each were studied: Vehicle group (DMSO), NSK-01105 group (60 mg/kg) and sorafenib group (60 mg/kg). |
| Experimental procedures | LNCaP and PC-3 cells were subcutaneous injected into the right flank of each animal. LNCaP and PC-3 tumors grew to about 120 mm^3^ in size on Day 10 or Day 12, respectively. Mice were grouped according to tumor size. Mice were treated orally once daily with vehicle (DMSO), NSK-01105 (60 mg/kg) or sorafenib (60 mg/kg) for 14 days. Tumor dimensions and body weights were recorded twice weekly. Animals were sacrificed under anesthesia after the last treatment and tumors were harvested and fixed in paraformaldehyde for immunohistochemical staining assay. The areas of CD31 positive objects were quantified. |
| Experimental  animals | Male Balb/c *nu/nu* nude mice (17.8 ± 1.2 g), aged 6-8 weeks, were included (n=36). |
| Housing and husbandry | Animals were maintained in laminar-flow cabinets under controlled environment at 25± 1 °C and humidity (55± 5%) on a 12-hours light/dark cycle and provided free access to food and water. All cages contained wood shavings, bedding and a cardboard tube for environmental enrichment. |
| Sample size | Thirty six healthy mice were divided into six groups of six each. |
| Allocating animals to experimental groups | LNCaP and PC-3 cells xenograft models include three groups of six each, respectively.  Mice were treated orally once daily with vehicle (DMSO), NSK-01105 (60 mg/kg) or sorafenib (60 mg/kg) for 14 days. |
| Experimental  outcomes | Two primary outcome measures were analyzed: overall performance on the Tumor volume and tumor weight. In addition, two secondary outcome measures were evaluated: side effects (including weight loss or death) and terminal performance in the microvessel area. |
| Statistical methods | The tumor weights were analyzed using one-way analysis of variance followed by Dunnett's test. Data points were presented as mean ± S.D.. For each test, the experimental unit was a group of animals. |
| RESULTS | |
| Baseline data | The animals’ health status was monitored throughout the experiments by a health surveillance programme according to Shenyang Pharmaceutical University Laboratory Animal Science Department guidelines. |
| Numbers analysed | Six animals in each group (6/6) were included in tumor weight analysis except for four animals in sorafenib treatment group in LNCaP xenograft models (4/6). Two animals were dead because of the toxicity of sorafenib (serious weight loss) on Day 6 and Day 11 after administration of sorafenib. |
| Outcomes and estimation | The effect of NSK-01105 in prostate cancer xenograft models.  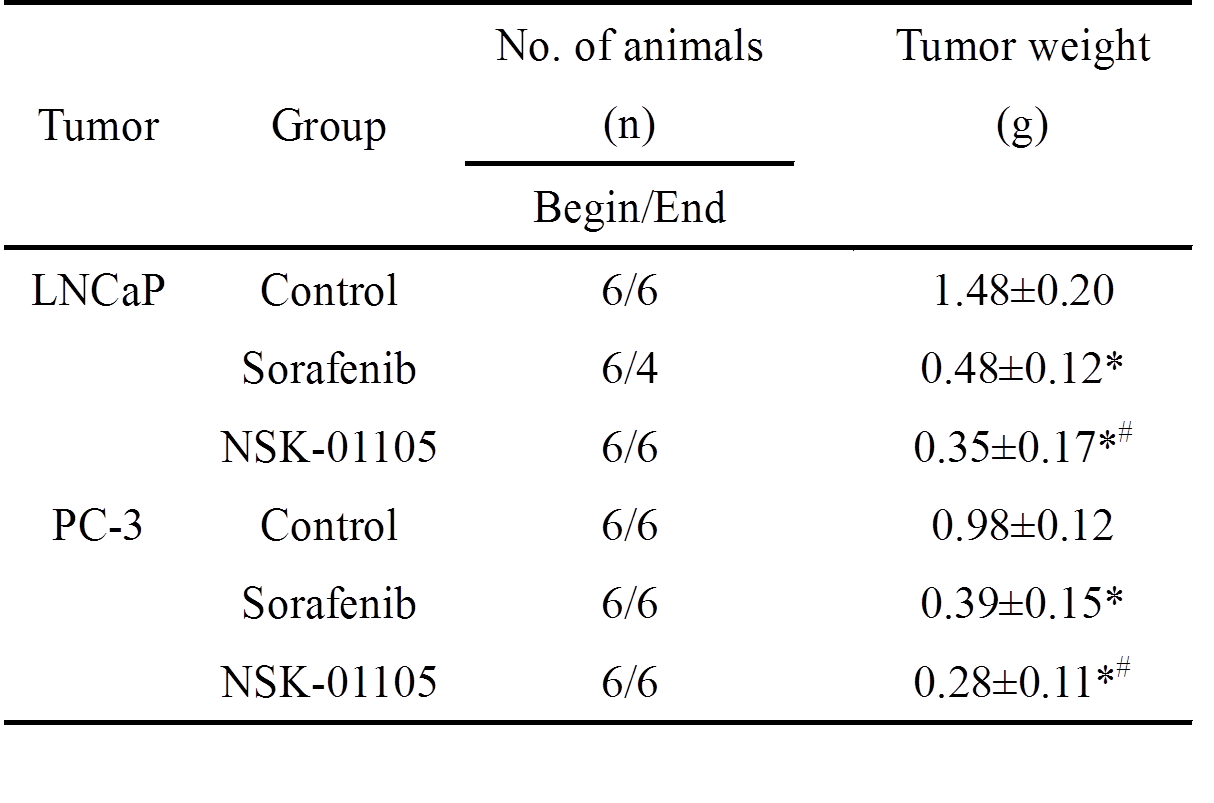  *, p < 0.05, compared with control. #, p < 0.05, compared with sorafenib.  NSK-01105 had more satisfactory inhibition effect against LNCaP and PC-3 tumor growth but with fewer side effects comparing to sorafenib. |
| Adverse events | Some side effects were observed in sorafenib group, including weight loss (10/12) or even death (2/12). In comparison, animals in NSK-01105 group only showed slightly weight loss (6/12). |
| DISCUSSION | |
| Interpretation/scientific implications | The aim of this study was to evaluate the effects of NSK-01105, a novel sorafenib derivative, on the inhibition of tumor specific angiogenesis in human prostate tumor xenograft models in order to support further drug development. Both VEGFR and EGFR are well known to regulate cell proliferation, differentiation, angiogenesis, and survival. Over-expression of both receptors is associated with cancer progression, poor prognosis or development of androgen independence. In our study, we confirmed that NSK-01105 significantly inhibited VEGF induced migration and tube formation of HUVECs at non-cytotoxic concentrations and inhibited angiogenesis in matrigel plug assay. NSK-01105 also inhibited viability and invasion of LNCaP or PC-3 cells. We have further demonstrated that the antiangiogenic activities of NSK-01105 were associated with reducing the VEGF-induced phosphorylation of VEGFR-2 and the activation of EGFR. Furthermore, NSK-01105 exhibited more satisfactory inhibition effect on tumor growth but with fewer side effects comparing to sorafenib in both xenograft models. |
| Generalisability/translation | Establishing anesthesia-independent settings for probing nude mice to the human prostate tumor cells are important for facilitating the detection of therapeutic targets at the cellular level. Knowledge of these targets is likely to help guiding the development of drugs for treating human prostate tumor. |
| Funding | This research was supported by National Natural Science Foundation of China No.81473188 (JT) and No. 81202038 (HW). |
